# Editorial: The origination of genetic novelties: New genes, new regulations, and new cell types

**DOI:** 10.3389/fgene.2022.1118926

**Published:** 2023-01-04

**Authors:** Wei Zhang, Li Zhang, Wenyu Zhang

**Affiliations:** ^1^ State Key Laboratory of Protein and Plant Gene Research, School of Life Sciences, Peking-Tsinghua Center for Life Sciences, Academy for Advanced Interdisciplinary Studies, Peking University, Beijing, China; ^2^ Chinese Institute for Brain Research, Beijing, China; ^3^ School of Ecology and Environment, Northwestern Polytechnical University, Xi’an, China; ^4^ Department of Evolutionary Genetics, Max Planck Institute for Evolutionary Biology, Plön, Germany

**Keywords:** regulatory mechanisms, genome evolution, phenotypic evolution, genomics, genetic novelty

## Introduction

Evolutionary novelties and new functions are fascinating. They play a substantial role in adaptive evolution and contribute to biodiversity but are also subject to developmental constraints and evolvability. The origination of novelties can occur at different levels, such as those of genes, genomes, and organisms. Subsequently, multiple pathways can lead to the origination of novelties, such as new genes, new regulatory mechanisms, new cell types, and new phenotypes. The rapid development of sequencing technologies and genome editing approaches allows such issues to be investigated in model and non-model organisms at the scales of gene evolution, cell type evolution, genome evolution, and phenotypic evolution.

Do different organisms gain new functions in a similar manner? How are multiple levels of novelties integrated, and how do they function? Research on new genes, new regulations, and new cell types will be needed to address their emergence and contribution to evolutionary novelties and new functions. We hope this Research Topic can help answer these key questions by integrating state-of-the-art analysis methods, such as comparative genomics, population genomics, single-cell transcriptomics, bulk transcriptomics, proteomics, comparative morphology, genome editing, and imaging. Therefore, the focus of this Research Topic is about aspects of gene evolution, cell type evolution, and evo-devo in a variety of organisms. We summarize the important findings described in this article Research Topic below.

## Exploring genetic novelties in model organisms

The Notch signaling pathway plays a key role in animal development and can be regulated by the protein ubiquitination of its major signaling components. Zhang et al. focused on the ubiquitin conjugating enzymes (E2 enzymes) involved in Notch signal transduction and demonstrated that the E2 enzyme *UbcD1* is required for Notch signaling activation during *Drosophila* wing development, which may play a positive regulatory role in Notch signaling by participating in the endocytic transport of Notch proteins, thus revealing a new role for *UbcD1* in developmental regulation.

Sequences that are transcribed but not translated, referred to as the “dark transcriptome,” are likely to play a non-negligible role in biological activities. Li et al. conducted an integrated study using multiomics data from *Saccharomyces cerevisiae* and identified a large number of unannotated but highly transcribed open reading frames that may be translated and encode species-specific proteins. In addition, this study integrated a project in which RNA-seq data was aggregated into the MetaOmGraph tool for interactive analysis and visualization of data from related studies, providing a basis for the further application of these public data for functional studies.

As a core component of the JNK signaling cascade, *Map2k7* is a conserved regulatory kinase gene that has a critical function in cell differentiation. Heinen et al. focused on the complex transcriptional patterns of this gene in mice and identified a novel testis-specific transcript of *Map2k7* driven by a new promoter in the house mouse subspecies *Mus musculus domesticus*, but the new promoter was lost secondarily in two other subspecies. The present study also demonstrated the contribution of this new promoter to sperm motility and the sperm transcriptome, revealing the functional innovation of a highly conserved gene.

The domestic silkworm synthesizes large amounts of silk proteins in its silk gland to produce cocoons and can thus be used as an ideal bioreactor for the large-scale production of recombinant proteins. However, genetic drift and random insertions due to transposon-based transformation prevent the stable expression of recombinant proteins. Li et al.established a natural *Sericin1* expression system to achieve the stable bulk expression of recombinant proteins and applied this system to produce significantly greater amounts of chimeric Serine1-EGFP as well as a human epidermal growth factor in cocoon shells than could be achieved using conventional transgenesis, providing a new strategy for using silk glands as bioreactors to produce recombinant proteins.

## Exploring genetic novelties in non-model organisms

*Papilio* butterflies exhibit polymorphic pupal colors and can therefore be used to study phenotypic plasticity. He et al. characterized expression profiles during the pupal development of *Papilio xuthus* using transcriptomics and proteomics. Instead of identifying genes related to the known pupal-cuticle-melanizing-hormone associated with brown pupal coloration, they identified genes in the neuropeptide cascade, genes related to the Toll signaling pathway and juvenile hormone, and cuticular proteins involved in pupal color formation, thus providing new ideas for studying butterfly protective coloration and phenotypic plasticity.

In bumblebees, queen mating triggers complex physiological and behavioral changes. Guo et al. profiled gene expression in queen spermathecae that were used for sperm storage and reproduction and found that queen mating caused differential expression of a large number of genes whose functions were mainly related to the immune response and sperm storage, showing that the transfer of external sperm into spermathecae resulted in upregulation of immune response genes. This study deepens the understanding of the postmating regulatory network in bumblebee queens.

The eastern oyster (*Crossostrea virginica*) is susceptible to Dermo, a fatal infection caused by the protist *Perkinsus marinus*, whereas the Pacific oyster (*Crassostrea gigas*) is resistant to Dermo. Chan et al. studied the interaction between these two oyster species and the parasite and identified dynamic and coordinated regulation of innate immune response genes. They identified *inhibitors of apoptosis* (*IAPs*) as an active defense mechanism in resistant oysters, greater expansion of *Toll-like receptors* (*TLRs*) in susceptible oysters, and positive selection for responsive *TLRs* in *P. marinus*, revealing the evolutionary history of interactions between oysters and parasites.

## Summary

In summary, the studies presented under this Research Topic involve a wide range of research interests and were conducted in a variety of research systems, focusing on both model organisms and non-model organisms. These studies have investigated the generation of novel functions in different developmental stages and biological activities at the gene, genome, and regulation levels, providing not only theoretical explorations but also important tools and methods and therefore expanding our knowledge and understanding of the origin of novel functions.

